# Building and Beta-Testing Be Well Buddy Chatbot, a Secure, Credible and Trustworthy AI Chatbot That Will Not Misinform, Hallucinate or Stigmatize Substance Use Disorder: Development and Usability Study

**DOI:** 10.2196/69144

**Published:** 2025-05-07

**Authors:** Adam Jerome Salyers, Sheana Bull, Joshva Silvasstar, Kevin Howell, Tara Wright, Farnoush Banaei-Kashani

**Affiliations:** 1 Clinic Chat, LLC, 2950 Arkins Ct, Unit 605, Denver, CO, 80216, United States, 1 3038079800; 2 University of Texas Health Sciences at San Antonio, San Antonio, TX, United States; 3 Department of Computer Science and Engineering, University of Colorado, Denver, CO, United States

**Keywords:** artificial intelligence, chatbot, infrastructure, substance use disorder, digital health, health communication, conversational agent, HIPAA, AI, Healthcare Insurance Portability and Accountability Act

## Abstract

**Background:**

Artificially intelligent (AI) chatbots that deploy natural language processing and machine learning are becoming more common in health care to facilitate patient education and outreach; however, generative chatbots such as ChatGPT face challenges, as they can misinform and hallucinate. Health care systems are increasingly interested in using these tools for patient education, access to care, and self-management, but need reassurances that AI systems can be secure and credible.

**Objective:**

This study aimed to build a secure system that people can use to send SMS with questions about substance use, and which can be used to screen for substance use disorder (SUD). The system will rely on data transfer via third party vendors and will thus require reliable and trustworthy encryption of protected health information .

**Methods:**

We describe the process and specifications for building an AI chatbot that users can access to gain information on and screen for SUD from Be Well Texas, a clinical provider affiliated with the University of Texas Health Sciences Center at San Antonio.

**Results:**

The AI chatbot system uses natural language processing and machine learning to classify expert-curated content related to SUD. It illustrates how we can comply with best practices in HIPPA (Health Insurance Portability and Accountability Act) compliance in data encryption for data transfer and data at rest, while still offering a state-of-the-art system that uses dynamic, user-driven conversation to dialogue about SUD, screen for SUD and access SUD treatment services.

**Conclusions:**

Recent calls for attention to user-friendly design concerning user rights that honor digital rights and regulations for digital substance use offerings suggest that this study is timely and appropriate while still advancing the field of AI.

## Introduction

Given the ongoing opioid epidemic in the United States [1], consideration of how and whether we can use digital tools to facilitate a response is warranted. One way that digital solutions may be deployed is by using them to scale screening for substance use disorder (SUD). Screening for SUD is critical to identify people at risk [2], as it can lead to referrals for services including outpatient as well as medically managed intensive inpatient services [3]. Although Screening, Brief Intervention, and Referral to Treatment (SBIRT) programs have been widely used to facilitate screening [4], and these programs are available electronically [5], there is still a major gap in screening, given current estimates indicate fewer than 10% of those at risk for SUD ever screened. This exacerbates an already costly response to the opioid epidemic, given that the United States spends more than $271 billion annually to address SUD-related health issues, crime, and lost productivity [6]. If we could enroll more people at-risk in treatment, we would save $4 for every treatment dollar expended [7].

Artificially intelligent (AI) chatbots that deploy natural language processing (NLP) and machine learning (ML) are becoming more common in health care to facilitate patient education and outreach [8,9]. These systems advance earlier generation rule-based chatbots that rely on “fixed state” messages that force users to choose from a predetermined set of responses to an interactive, user-driven system where people can initiate conversations on any number of topics and chatbots can generate answers on-the-fly. The current AI chatbots, particularly generative chatbots such as ChatGPT [10], rely on access to a large language model (LLM) for training bots to correctly classify and respond to queries. These LLMs represent a much more sophisticated way to interact with people that allows for rapid exploration of vast data sources to learn how to correctly understand the intention of the queries that people make, to make inferences from data, and generate relevant answers in response to user queries.

There is an ample body of evidence from investigations of earlier, fixed-state message systems showing they can be used effectively to positively impact health behaviors and health outcomes [11]. Although we do not yet know the impact of moving from fixed-state chatbots to conversational and generative AI systems, health care systems that have adopted these AI systems are optimistic that they will offer ample return on investment [12]. Since the more advanced AI systems are nascent, we have not yet established standards for best practices in their design. System users and developers of AI chatbots, including generative chatbots such as ChatGPT, have raised concerns related to their use that warrant attention for health-related applications [13] Ethical concerns include observations that systems could discriminate against stereotype or stigmatize users, and could also compromise privacy and data sovereignty [14,15]. Researchers have documented tendencies for generative chatbots to misinform [16], hallucinate [17], and obscure information about how data are being accessed and used [18].

Recent reviews and published literature have highlighted the critical challenges that are inherent in maintaining security for AI chatbots. They highlight chronic concerns with data breaches and malicious input and called for standards such as end-to-end encryption, organizational control, and adversarial training (ie, purposefully attempting to confuse a system during development to train it to recognize potentially malicious inputs) to mitigate these [19,20]. One researcher has highlighted additional critical concerns that are specific to health care organizations that seek to use AI chatbots, including a need to align chatbot design and security with Healthcare Insurance Portability and Accountability Act (HIPAA) regulations that govern patient protections in care delivery [21].

Optimizing digital tools for substance use is warranted. However, we currently do not have digital tools that focus explicitly on user rights, including privacy; are evidence-based; user friendly; easily accessible, person-centered [22]; and can be delivered without generating or reinforcing stigma.

In this paper, we present the infrastructure and technical specifications used to design Be Well Buddy, an AI chatbot focused on raising awareness and access to screening and treatment for SUD. It intentionally addresses the security and ethical concerns identified here. We also present findings from a beta test of the system whose goals were to verify system security and functionality.

## Methods

This work represents a partnership between Clinic Chat, LLC, a health technology start-up company, and The University of Texas Health Sciences Center at San Antonio (UT Health San Antonio), a US-based university with a robust clinical program called Be Well Texas, focused on screening, treatment, and social support for SUD. The principals from Clinic Chat have a background in scientific research related to health as well as the technical skill to design and deploy AI chatbots; UT Health San Antonio and Be Well Texas have a wide-reaching health education initiative focused on substance use prevention and treatment. The partnership was established to adapt health AI chatbots to focus specifically on raising awareness and providing access to screening for and treatment of SUD within the university program.

### System Specifications

Given concerns that generative chatbots can hallucinate and misinform, along with ample evidence that careful attention to the design of messages for health communication can be more impactful than generic messaging, we determined it appropriate to develop and curate messages specific to SUD that could be delivered by a closed-domain AI chatbot system (ie, one where messages returned in response to user queries would only come from this specific library of messages). This approach allows us to avoid a common pitfall of generative AI such as ChatGPT that can reinforce biases or misinform when seeking responses to user queries. By using a closed library of responses that have been reviewed for accuracy, empathy and tone, our system can only choose from a limited group of options that are medically correct and consistent with clinical guidelines for care. Guided by literature documenting approaches to health communication that can increase engagement with messages [23], we generated content that allowed for tailoring to individual users to increase content relevance (eg, by prefacing each message with their first name or by inviting them to name and explore preferred topics) [24]; that could offer intuitive suggestions for behavioral decision-making [25,26]; that offered narrative, emotional messaging, status-enhancing and skill-building content [27]; and that worked to destigmatize substance use [28]. These message design strategies have consistently been shown to have a positive impact on health behavior [29-31]. We developed an initial library of messages and then reviewed the message content in a formative process with members of the intended audience of the chatbot to obtain their feedback on the message content, tone, acceptability, and capacity to engage people in dialogue. One key outcome from this process was the name “Be Well Buddy,” which we gave to the system, generated from a participant in this formative message development process (Sarah Mumby, MPH, email August 15, 2024).

### Beta Testing

Once the system was designed and built, we conducted a one-day beta test to ensure system functionality with members of the study team from Clinic Chat and UT Health San Antonio. All participants were encouraged to try and push the system to its limits. Specifically, they asked expected questions (eg, What is a substance use disorder? What is medication-assisted therapy? How much does treatment cost?) and asked multiple questions in a short period of time (ie, five-six questions in a minute). They made queries in different languages and completed all the screeners embedded in the system. They deliberately worked to confuse the system with questions that were nonsensical or unrelated to SUD. Our goals for the beta test were to (1) identify errors in sending and receiving messages and use this to determine the system’s precision (ie, whether responses sent by the system appropriately matched the intent behind the user queries); (2) identify any problems with encryption; and (3) identify any delays in message delivery.

### Ethical Considerations

The work we describe here was reviewed by UT Health San Antonio Institutional Review Board, who deemed these activities as preliminary to research conducted exclusively by paid research staff and therefore exempted from requirements for human subjects’ approval. Identifying data for persons beta-testing the system included telephone numbers, which were encrypted once outside the UT Health San Antonio firewall. The data presented in this paper informed an observational study that has been reviewed and approved by the UT Health San Antonio Institutional Review Board (protocol number 20230662H).

A description of system specifications and functionality, and results of the beta test is given below.

## Results

### System Specifications

**Figure 1.**
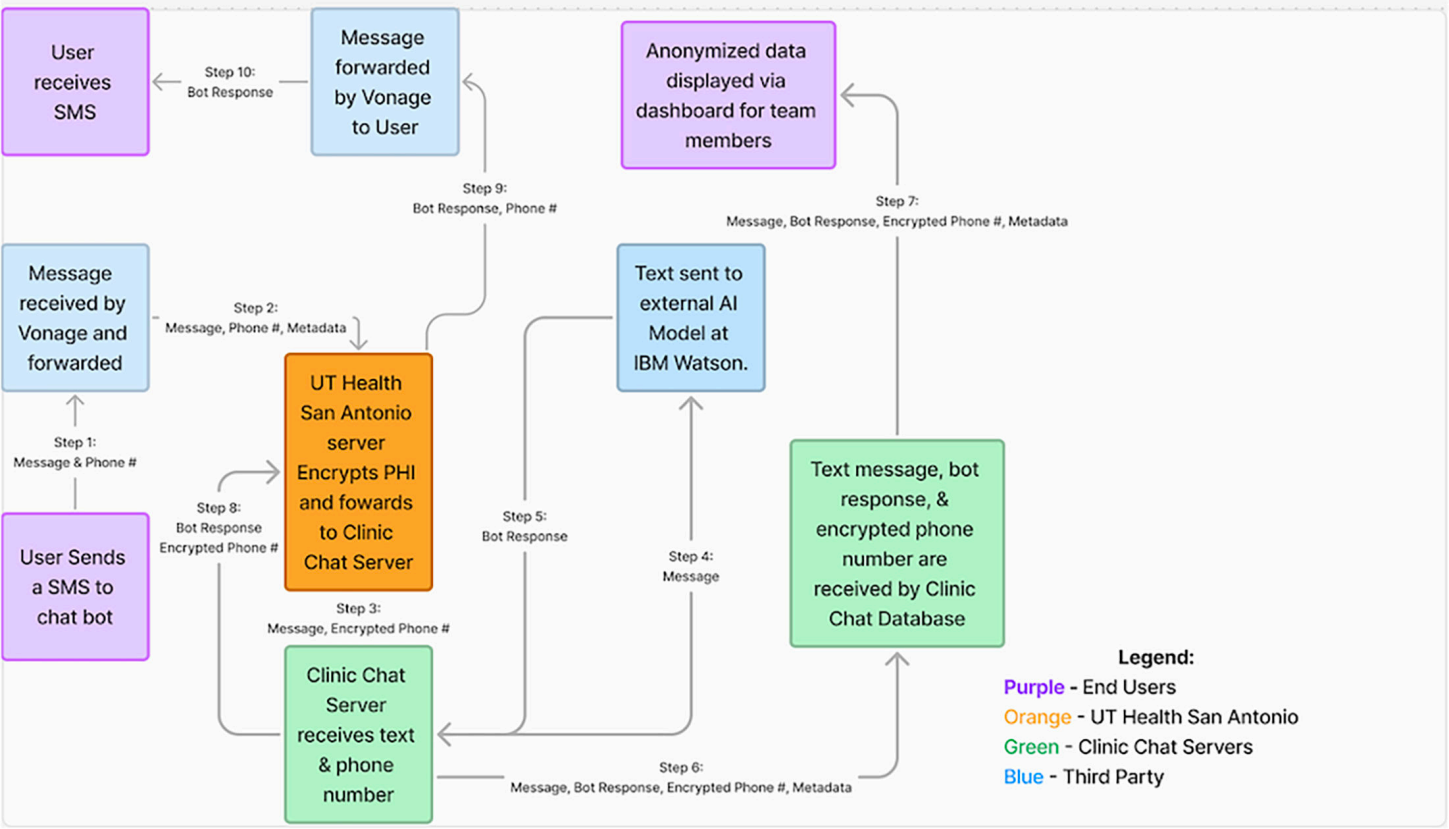
System workflow. AI: artificial intelligent; PHI: protected health information.

Figure 1 illustrates how the system retrieves and shares information, along with a description of the security steps that are in place to ensure user data are protected. In this figure, purple boxes represent end users of the system, ie, people who send and receive text messages via SMS. The blue boxes represent any third-party vendor that retrieves or sends information from the user to the Clinic Chat AI Chatbot, called “Be Well Buddy.” The orange box represents the firewall for UT Health San Antonio, home to Be Well Texas, the clinical entity whose patients and prospective patients are the intended users of Be Well Buddy.

Data exchanges between users and the AI chatbot are described as steps in Figure 1. In Step 1, Be Well Buddy initiates a dialogue with users when it sends an initial SMS message to them after receiving information on their first name and telephone number from UT Health San Antonio, which they provide upon enrolling a user in the feasibility study. All SMS messages—incoming and outgoing—are handled by our third-party provider, Vonage Telecommunications Company (Step 2).

System users can respond to any message from Be Well Buddy. When this is done, the data are forwarded to the UT Health San Antonio Server (Step 3), where we host a Flask application designed for minimal impact on system performance. This Flask app uses a webhook to ingest the information efficiently. The lightweight nature of Flask, as opposed to heavier frameworks like Django, is crucial in maintaining fast processing speeds behind the secure firewall. The information received by the Flask app includes one piece of protected health information (PHI)— the phone number—which is now encrypted using SHA-256 bit encryption. This robust encryption method ensures that the data cannot be read or altered without the encryption key and remains encrypted anywhere outside the firewall, including Clinic Chat Amazon Web Server (AWS).

The message content and metadata without identifiers (eg, message time, type), along with the encrypted phone number are sent to a Clinic Chat-owned and managed AWS, known as EC2 instance that hosts a Django application to authenticate and retrieve information from the Be Well Buddy chatbot; only whitelisted IP’s can send requests to the AWS EC2 instance.

At this point, a request is made to our IBM Watson AI (version 10.2.0) assistant that includes only the message body from the SMS message (Step 4). IBM Watson currently serves as our NLP AI system to classify message content. Prior to forwarding messages to Watson, our Django app (version 5.2) performs a variety of preprocessing tasks using regex and custom code to effectively manage number inputs, survey and screener responses, and any content that does not constitute a direct user query. While IBM Watson effectively classifies message content and returns a response (Step 5), we plan to transition to an internally hosted LLaMA integration in the future to leverage the impressive capabilities of LLMs. This response, along with the encrypted phone number, is sent back to a Flask app—a lightweight application optimized for faster processing—hosted behind the university server before being forwarded to the user through Vonage.

The response, message, metadata, and encrypted phone number are sent to an Amazon Web Server (AWS) Relational Database Service (RDS) instance for storage (Step 6). A dashboard hosted on the Clinic Chat subdomain (through another AWS EC2 instance) can then send requests for the system to retrieve data so team members can view large or small trends in anonymized message data (Step 7).

The system relies on data transfers with multiple third-party vendors, including Vonage that aggregates message delivery via multiple cell phone providers; IBM Watson, which classifies message content; and AWS that stores data. Following is a description of how each of these providers operate with the Be Well Buddy chatbot.

Vonage is our third-party telecommunications provider. They manage all outgoing and incoming messages. As they handle phone numbers, which are considered PHI that is regulated under the HIPAA, they have executed a Business Associate Agreement with Clinic Chat. This Business Associate Agreement ensures that both Vonage and Clinic Chat remain compliant with HIPAA regulations related to PHI by redacting all phone numbers and messages from Vonage logs. To retrieve the phone numbers and message information, the UT Health San Antonio server has generated a whitelist of IP addresses from Vonage (ie, the IP addresses for computers used by Clinic Chat staff) that will limit JSON data intakes exclusively from these IP addresses. All data will be sent to Vonage and received from Vonage through HTTPS, a secure data transfer protocol that encrypts data during transfer.

IBM Watson is used t0 store data to train our AI models, allowing the system to more precisely interpret incoming queries from users so the system can then respond with a message from our curated library that corresponds to the intent behind each user query. IBM Watson will never receive any PHI in any form from messages—they only receive user queries as the text message body, which is used to match to one of our answers in our library of responses before returning it to the Clinic Chat AWS instance [32] (Step 8).

We are using two AWS EC2 server instances—one to host the backend server responsible for connecting the UT Health San Antonio server, IBM Watson and the database, and a second to host the front-end team interaction and database. Both are configured according to AWS HIPAA guidelines, although unencrypted PHI will never reach AWS. The backend server will only connect to the UT Health San Antonio server via HTTPS connection, IBM Watson, and AWS RDS. We whitelist the UT Health San Antonio server IP (and a few developer IPs for testing purposes), but no other IPs will be able to hit the server. The front end is also whitelisted to limited team member IPs and contains a login and user authentication system to view anonymized data.

We also use an AWS RDS instance to store our data for the project. All PHI are encrypted when entering AWS and are stored in encrypted form in RDS; they are never decrypted in AWS. The RDS database is connected to EC2 instances. The backend will only write data to the database, and the front end will only pull data.

The UT Health San Antonio server hosts a Flask app to process and encrypt PHI before passing it on to AWS, and process and decrypt phone numbers when sending it to Vonage. The app has a webhook that interprets a series of whitelisted IPs from Vonage, which will send JSON data including phone number and message content. Phone numbers are the only PHI the system receives, so the Flask app encrypts the phone number using Python Fernet encryption before sending the message content and encrypted phone number to our AWS EC2 backend instance. Additionally, the Flask app has a webhook that receives incoming JSON data from the same AWS EC2 instance. The AWS EC2 instance is whitelisted, and along with Vonage, these are the only servers that are whitelisted (outside of a dedicated IP for a developer to fix any issues). The Flask app will take in an encrypted phone number and response message from the EC2 instance, decrypt the phone number, and pass the phone number and response message back to Vonage to be sent to the user (Steps 9 and 10).

In anticipation of possible system attacks, we also built in protocols to reduce their impact should they occur. We exposed our model to anticipated queries that are adversarial (eg, “I don’t want to screen for SUD, leave me alone,” or “Your answers are stupid”) to train for resilience and respond professionally (eg, “I’m sorry you are disappointed with my response. I would be happy to try again or discuss a different topic”). When a user asks the system a question, the response is chosen from the library using NLP and then relies on probabilistic models to determine how likely it is that a response from our library will match the intent behind the user query. We monitor all logs to determine if system users and system content are behaving as expected and to identify any suspicious interactions.

### Beta Testing

The beta test was conducted over the course of one day with six users. Users sent 426 messages to the system (for an average of 71 messages per user, with a range of 25‐110 messages), and the system responded 800 times. The additional system responses included intentional follow-up prompts (eg, “Ask me about something else! I can answer your questions about medication-assisted therapy” or “Are you interested in screening for SUD?”) that were appended to responses. We documented two instances where users received responses in Spanish, although the content in the library was only available in English. Upon investigation, we determined that the system was retrieving messages from a different library within the Clinic Chat system focused on chronic illness self-management. We unlinked the libraries between the SUD and chronic conditions content to avoid this error in the future. These two errors were the only ones we documented when the system returned an incorrect response (ie, 2 of 426 or <1%). When a user sent a nonsensical query or one that did not align with SUD, the system correctly responded with “I’m sorry, I am still learning and did not understand your question. Will you please ask again in different words?” Thus, we established a >99% level of system precision overall. We documented three instances where message return was quite slow (ie, a >90 seconds to return a response). In all other instances, responses were sent within the first 10 seconds of message receipt. We recognized this as a problem with Vonage and reached out to report the message delays to them. We identified a problem with the responses returned to users who screened for anxiety, depression, and SUD, where those with high risk were not alerted to their risk and given a referral, necessitating a review and correction of the referral algorithm. The algorithm for screening and responses related to screening were incorrect and fixed. We did not identify any problems with system encryption or data security in transferring queries via SMS. Given the short time frame and small sample of beta testers, we did not experience any adversarial attacks to our system during the beta test and cannot report on the robustness of our protocol to address these attacks in this paper.

## Discussion

In this study, we present the technical specifications included in Be Well Buddy to facilitate access to information and screening for SUD for Be Well Texas, a clinical organization affiliated with the UTHSA. Our system adheres to HIPAA regulations for the protection of PHI while avoiding pitfalls of current generative AI chatbots by using curated chat content that does not misinform or hallucinate. Users of Be Well Buddy can get credible and complete information about SUD and can access care on a 24/7 basis. The beta test uncovered and resolved two functional errors; ie,(1) delivery of messages unrelated to SUD and (2) an incorrect algorithm for screening feedback. After correcting these errors, we verified that the system functions as intended and has a high level of precision [33].

This study is not without limitations. Our objective was to ensure that the system worked without error and was secure; therefore, using a small number of beta testers was appropriate to achieve these findings [34]. However, the small number of beta testers did not allow us to delve deeply into how well the content resonated for users. Further, as noted above, given the short time frame and small sample of beta testers, we did not experience any adversarial attacks to our system during the beta test and thus cannot report on the robustness of our protocol in such scenarios. These topics would be appropriate for a subsequent trial of system use.

While we stand behind our approach of using a closed library system to avoid challenges with misinformation and hallucination, this model requires regular updates to content to ensure it remains consistent with medical and professional guidelines related to SUD, SUD screening and treatment referrals. This task may evolve to become cumbersome if library updates are frequent or extensive.

AI chatbots can struggle with delineating between nuanced concepts, making it difficult to support complex questions from users. As we rely on IBM Watson for classification and other language models, this challenge persists and will require careful attention to identify inaccuracies to allow for appropriate reclassification of content when systems do not respond with precision.

Finally, we recognize that the datasets used to train AI chatbots have inherent biases, which we risk reproducing when scaling this system. This risk can be particularly challenging for the Be Well Buddy chatbot whose explicit goal is to reduce stigma. Extra effort is recognized to ensure that the content does not produce or reinforce stereotypes about substance use or people who use substances [35].

According to a recent McKinsey report [12], a large group of surveyed health care leaders indicated that their organizations are eager to use generative AI to enhance operations—particularly patient engagement processes—but most are still adopting a wait-and-see approach. While traditional rule-based chatbots have already proven effective, the AI-driven chatbots, particularly those that are driven by generative models such as ChatGPT, have yet to be proven safe for health care applications. In particular, a number of ethical concerns, such as privacy, confidentiality, bias and fairness, transparency, accountability, regulatory compliance, risk anticipation and copyright complications remain the subject of active research and mitigation in generative models. Additionally, the risks of misinformation, perpetuation of bias, or hallucination continue to dampen enthusiasm for widespread deployment of AI Chatbots in health care [8].

Until effective and proven solutions are developed to address these concerns, widespread use of AI-driven chatbots in health care applications is not anticipated.

In this paper, we introduced the Be Well Buddy chatbot, a novel AI-driven chatbot that adopts a secure and reliable approach to engage with patients with SUD. Our system circumvents challenges with misinformation, bias, and hallucination, while securely delivering and supporting access to SUD screening and treatment options.

Our research is consistent with recent recommendations for optimized digital substance use interventions, such as solutions that emphasize digital rights of privacy and confidentiality, accessibility, and user-friendliness [15]. In the future, we will further experiment with Be Well Buddy to evaluate and report its efficacy in promoting self-screening and referral for SUD. We anticipate integration with treatment providers who wish to accelerate referrals for care by inviting persons living in their catchment areas to use the system and for self-screening. This system may also be integrated into other organizations adjacent to SUD treatment, such as the criminal justice system where care may be mandated, or the parole system where people may seek to closely monitor their own or clients’ risk for SUD.

## Abbreviations

AI: artificial intelligence
AWS: Amazon Web Server
HIPAA: Healthcare Insurance Portability and Accountability Act
LLM: large language model
ML: machine learning
NLP: natural language processing
PHI: protected health information
SUD: substance use disorder
